# Reappraisal of profibrotic phenotype and cell-cycle state of renal tubular epithelium after ischemia–reperfusion injury

**DOI:** 10.1371/journal.pone.0347835

**Published:** 2026-04-24

**Authors:** Daichi Fukaya, Wakako Kosakai, Tetsuya Sato, Tsutomu Inoue, Hiroaki Amano, Takeru Kusano, Hirokazu Okada

**Affiliations:** 1 Department of Nephrology, Faculty of Medicine, Saitama Medical University, Saitama, Japan; 2 Faculty of Medicine, Biomedical Research Center, Saitama Medical University, Saitama, Japan; 3 Faculty of Medicine, General Internal Medicine, Saitama Medical University, Saitama, Japan; University of Bergen, NORWAY

## Abstract

Chronic kidney disease progression involves phenotypic changes in tubular epithelial cells, and recent studies have highlighted the relationship between such pro-fibrotic phenotypes and cell cycle arrest in injured tubular epithelial cells undergoing repair. We investigated these processes using a mouse unilateral ischemia–reperfusion injury model with γGT.Fucci2aR mice, which express fluorescent cell cycle markers, and *in vitro* experiments with human kidney-2 cells and public single-cell RNA sequencing data. In the unilateral ischemia–reperfusion injury model, mVenus-positive cells (S/G2/M phases) in the γGT.Fucci2aR mice peaked on Day 3 post-injury, then rapidly declined. In kidneys with progressive tubular atrophy and interstitial fibrosis between 7–12 days post-injury, S/G2/M phase cells were limited. These findings were corroborated by analysis using public single-cell RNA sequencing data from the same mouse models, which confirmed dynamic cell cycle changes in the acute phase post-injury but no significant G2/M phase cells in the chronic phase. *In vitro* experiments with human kidney-2 cells demonstrated that cellular communication network factor 2 and transforming growth factor-β expression increased significantly as proliferating cells reached confluence and cell cycle progression slowed. Pro-fibrotic phenotypes in tubular epithelial cells were not exclusively acquired by G2/M-arrested cells, as reported in previous studies, but can also be acquired by cells in the quiescent G0/G1 phase during normal cell cycling. To develop novel therapeutics for chronic kidney disease, regulating pro-fibrotic gene expression in injured tubular epithelial cells, independent of specific cell cycle phases, appears to be crucial.

## Introduction

Chronic kidney disease (CKD), a chronic loss of kidney function with a glomerular filtration rate of <60 mL/min per 1.73 m^2^ over 3 months, is the 14th-leading cause of death worldwide. Its significance lies not only in requiring renal replacement therapy upon progression but also in constituting the underlying pathology for major causes of death (infections and cerebrocardiovascular diseases), excluding malignancies. Nonetheless, effective treatments for CKD are currently lacking. The incidence of CKD has increased dramatically since 1990 and is now a global concern [1]. Renal fibrosis is a pathological change commonly observed during CKD progression, regardless of the primary disease, with activated (injured) tubular epithelial cells playing a central role in its formation and progression [2–8]. Cellular communication network factor 2 (CCN2; previously called connective tissue growth factor) is one of the pro-fibrotic factors produced by tubular epithelial cells when stimulated by transforming growth factor-β (TGF-β). CCN2 is a matricellular protein involved in fibrosis in multiple organs. In the kidney, CCN2 binds to integrins on tubular epithelial cell surfaces and promotes renal fibrosis through phosphorylation of focal adhesion kinase. In mouse models, suppression of CCN2 expression in tubular epithelial cells inhibits renal fibrosis by approximately 70% following unilateral ischemia–reperfusion injury (UIRI) [2,3]. Therefore, CCN2 serves as a therapeutic target for CKD and marker molecule for evaluating renal fibrosis, and also as a pro-fibrotic factor expressed in tubular epithelial cells, similar to TGF-β.

Recently, the relationship between the cell cycle and pro-fibrotic gene expression in tubular epithelial cells has attracted attention. Under physiological conditions, renal tubular epithelial cells remain almost exclusively in the G0/G1 phase [9]. When severe damage, such as ischemic acute kidney injury (AKI) occurs, surviving tubular epithelial cells proliferate through cell division to replace necrotic and detached tubular epithelial cells, thereby contributing to renal functional recovery [10]. This cell cycle-controlled mechanism is the same as that of normal cell division. In cells with DNA damage, p21^Waf1/Cip1^, which has a cyclin-dependent kinase (CDK) inhibitory effect, is induced via p53, thereby arresting cells in the G1 phase. This is due to the checkpoint mechanism that aims to secure sufficient time for DNA damage repair [11]. In contrast, prolonged block in G1 after AKI increases TGF-β expression, resulting in a pro-fibrotic phenotype and potentially leading to fibrosis [12].

Previous reports on the association between cell cycle arrest and pro-fibrotic phenotype in renal tubular epithelial cells following AKI have shown variable results. Yang et al. found that TGF-β and CCN2 were highly expressed in tubular epithelial cells arrested in the G2/M phase after injury; they reported that tubular cells with failed repair remaining in G2/M phase may be associated with increased expression of TGF-β and CCN2 [8]. This finding provides a compelling mechanism for autonomous progression of renal fibrosis, where TGF-β and CCN2 expression does not decrease, even in the chronic phase after the acute inflammatory period has passed and inflammatory cell infiltration, including macrophages, has disappeared. However, there has been a discrepancy with the classical observation that pro-fibrotic factors, such as TGF-β, act on normal tubular epithelial cells independent of cell cycle arrest or DNA damage, thereby inducing epithelial-mesenchymal transition and increased CCN2 expression, leading to fibrosis. Although the contribution of G2/M arrest in CKD progression has gained attention, recent single-nucleus RNA sequencing by Gerhardt, et al. has challenged this view, showing no increase in G2/M-arrested cells in injured tubular epithelial cells stimulated by inflammatory cytokines with activated SMAD3, nuclear factor-kappa B, and activator protein 1 [13]. Thus, pro-fibrotic gene expression may be upregulated in various cell cycle states, including in G0/G1-arrested and proliferating cells.

The relationship between the cell cycle and gene expression of pro-fibrotic factors in tubular epithelial cells remains unclear. Here, we evaluated mouse models of UIRI using fluorescent labeling (Fucci2aR) [14]. This is to visualize the cell cycle of tubular epithelial cells during AKI to CKD transition and to examine the relationship between the cell cycle and CCN2 gene expression in cultured human renal tubular epithelial cells (human kidney-2 [HK-2]). Furthermore, we analyzed publicly available single-cell RNA sequencing (scRNA-seq) data to investigate cell cycle changes in tubular epithelial cells in the UIRI model.

## Materials and methods

### Animal model

Genetically modified mice (R26Fucci2aR; RBRC06511) were obtained from RIKEN BioResource Research Center (RIKEN BRC, Tsukuba, Japan) and were backcrossed ≥10 times with SJL/J mice before experiments to establish an SJL background. Polymerase chain reaction (PCR) genotyping was performed using KOD One PCR Master Mix (KMM-201; Toyobo, Osaka, Japan) with PCR primers and annealing temperature as follows: R26_WTF(RM), CAAAGTCGCTCTGAGTTGTTATC; R26_WTR(RM), GGAGCGGGAGAAATGGATATGAA; and F2AR(RM), TGGCGGCCGCTCGAGATGAATC, 60°C [14].

### HK-2 cell line

The cultured human renal tubular epithelial cell line HK-2 (ATCC Catalog Number: CRL-2190) was cultured in the Ham’s F-12 and Dulbecco’s Modified Eagle Medium (4.5 g/L glucose) (Nacalai Tesque, Kyoto, Japan) supplemented with 5% fetal calf serum, 100 U/mL penicillin, and 100 µg/mL streptomycin in a humidified atmosphere at 37°C with 5% CO_2_. Upon reaching confluence, the cells were seeded at a density of 1.1 × 10⁴ cells/cm^2^ and promptly passaged. For the experiments, HK-2 cells were seeded in six-well plates (1 × 10⁵ cells/well) and incubated for 24 h. After confirming normal cell adhesion, the cells were subjected to low-oxygen and low-nutrient conditions (1% O_2_ and 0% glucose for 24 h) (Thermo Fisher Scientific, CO2 incubator 3130, MA, USA) to synchronize (arrest) the cell cycle, designated as “Control” condition. The cells were then returned to normal conditions (20% O_2_ and 4.5% glucose) and observed up to 72 h. To examine the relationship between cell number/culture environment and CCN2 expression, we established two conditions: the standard seeding density described above (“Stnd”) and a high-density condition with double the cell number at seeding (“Hi”).

### Mouse models of CKD

We used F1 mice (hereafter referred to as gamma-glutamyl transpeptidase [γGT].Fucci2aR) generated by crossing R26Fucci2aR (Cherry-cdt1; mVenus-Geminin) mice, which enable fluorescence visualization of the cell cycle, with γGT.Cre mice (SJL background) [2]. In these mice, Fucci2aR expression is induced in cortical tubular epithelial cells with high γGT expression. Cdt1, a component of the transcription initiation complex, is highly expressed in the G1 phase of the cell cycle. In contrast, geminin is highly expressed in the S-G2 phase and directly binds to Cdt1, inhibiting Cdt1’s binding to the genome, thereby uniquely defining DNA replication. Therefore, in γGT.Fucci2aR mice, Cherry is highly expressed in the G1 phase of these cells and mVenus in the S/G2/M phase in cortical tubular epithelium only, allowing fluorescence visualization of the cell cycle.

Male mice aged 6–8 weeks were used to establish the UIRI model. For anesthesia, a mixture of medetomidine hydrochloride 1.0 mg/mL, midazolam 5.0 mg/mL, and butorphanol tartrate 5.0 mg/mL (0.1 mL/10gBW, intraperitoneal administration) was used. Under anesthesia, the left kidney was accessed via the lateral flank approach [2], and after processing the connective tissue at the renal hilum, the renal artery and vein were clamped for 30 min with metal clips (Mizuho Corporation Sugita Clip Catalog #07-940-81, Tokyo, Japan) while maintaining rectal temperature at 37°C using a thermoregulation system (temperature maintenance device [#69020], heating pad [#69024], and temperature probe [#69022], all from Neuroscience, Tokyo, Japan). After confirming restoration of renal blood flow upon clamp release, the incision was closed with 3−0 silk sutures, and the animals were kept warm until recovery from anesthesia. The same anesthetic mixture was used for organ harvesting.

This study was conducted in accordance with institutional regulations based on the “Basic Policies for the Conduct of Animal Experiments in Research Institutions” and “Saitama Medical University Animal Experiment Regulations,” as well as the “Saitama Medical University Recombinant DNA Experiment Safety Management Regulations” based on the “Act on the Conservation and Sustainable Use of Biological Diversity through Regulations on the Use of Living Modified Organisms (Cartagena Act)” (Approval numbers 3268, 3544, 3793, 4049).

### Morphological and fluorescence analyses

The kidneys were removed under anesthesia and immediately fixed with 4% paraformaldehyde, then paraffin-embedded blocks were prepared, sectioned at 4-μm thickness, and stained with the Masson’s trichrome stain for histological evaluation of renal fibrotic lesions. For fluorescence observation, the kidneys fixed with 4% paraformaldehyde were sectioned using a vibratome (Leica Vt-1200; Wetzlar, Germany) and cleared using the CUBIC method. CUBIC, defined as Clear, Unobstructed Brain/Body Imaging Cocktails and Computational analysis, is a tissue-clearing technique that renders organs transparent by removing lipids and homogenizing the refractive index, enabling three-dimensional visualization of intact tissue structures [15,16]. Observations were made using a confocal laser microscope (OLYMPUS FV1000, Tokyo, Japan). To count mCherry-positive and mVenus-positive cells, three sections were prepared from each kidney, and the cells were counted in 10 randomly selected fields in the cortical region observed with a 40 × objective lens. Owing to inter-individual differences in recombination efficiency, the ratio of mVenus-positive cells (numerator) to mCherry-positive cells plus mVenus-positive cells (denominator) within 10 fields was used as an index to represent “the proportion of tubular epithelial cells with dynamic cell cycles.”

### Real-time semi-quantitative reverse transcription PCR

Total RNA from the kidneys was extracted using TRIzol (Thermo Fisher Scientific, MA, USA). Real-time semi-quantitative reverse transcription PCR (RT-qPCR) was performed as previously described [3]. Briefly, mRNA levels were semi-quantified by one-step RT-qPCR using a QuantiTect SYBR Green RT-PCR kit (Qiagen, Hilden, Germany) and QuantStudio 12 K Flex system (Thermo Fisher Scientific), with glyceraldehyde-3-phosphate dehydrogenase (Gapdh) as an internal control. The relative expression levels of each mRNA on Days 3, 7, and 12 were evaluated, with the average expression level of each mRNA in normal kidneys set at 1.0. For the HK-2 cells, cDNA was synthesized using the ReverTra Ace qPCR RT Kit (Toyobo, Osaka, Japan). PCR was performed using Thunderbird Next qPCR Mix (Toyobo) and Step One Plus real-time PCR system (Thermo Fisher Scientific). The relative expression difference index (ΔΔCt value) of each mRNA relative to Gapdh was evaluated. Primer sets used are listed in Table 1.

**Table 1:** List of primer sets.

*Collagen type I α1*	*Col1a1*	AGACATGTTCAGCTTTGTGGAC	GCAGCTGACTTCAGGGATG	mouse
Fibronectin EIIIA isoform	*Fn-EIIIA*	ATCCGGGAGCTTTTCCCTG	TGCAAGGCAACCACACTGAC	mouse
CCN2	*Ccn2*	CTCTCGTCGCCTCTGCAC	CTGCAGTCCTGGCCCATA	mouse
		AATGCTGCGAGGAGTGGGT	CGGCTCTAATCATAGTTGGGTCT	human
Snail1	*Snail1*	CTTGTGTCTGCACGACCTGT	AGGAGAATGGCTTCTCACCA	mouse
Transforming growth factor-β1	*Tgf-*β	GAGGGCTGAACCAAGGAGAC	ATCCCGTTGATTTCCACGTG	mouse
		GCTACCATGCCAACTTCTGC	TATGCTGGTTGTACAGGGCC	human
Cyclin-dependent kinase 1	*Cdk1*	CCTAGCATCCCATGTCAAAAACTTGG	TGATTCAGTGCCATTTTGCCAGA	human
Cyclin-dependent kinase 5	*Cdk6*	GGATAAAGTTCCAGAGCCTGGAG	GCGATGCACTACTCGGTGTGAA	human
p21	*p21*	AGGTGGACCTGGAGACTCTCAG	TCCTCTTGGAGAAGATCAGCCG	human
Glyceraldehyde 3-phosphate dehydrogenase	*Gapdh*	TGCAGTGGCAAAGTGGAGATT	TTGAATTTGCCGTGAGTGGA	mouse
		CTCTGCTCCTCCTGTTCGAC	ACGACCAAATCCGTTGACTC	human

### Cell cycle analysis

Cultured cells were detached with 0.25% trypsin-ethylenediaminetetraacetic acid, washed with phosphate-buffered saline, fixed by adding 70% ethanol while vortexing, and stained with 4’,6-diamidino-2-phenylindole (2 µg/mL) (Thermo Fisher Scientific) in phosphate-buffered saline for 5 min at room temperature. DNA was analyzed by flow cytometry (Cell Sorter SH800Z, SONY, Tokyo, Japan) using 405 nm excitation, with ≥10,000 events collected per sample. Cell cycle distribution was determined using the built-in software of a flow cytometer (Cell Sorter SH800Z). Cell cycle analysis was performed by classifying cells based on each cell’s nuclear fluorescence intensity as quantified by flow cytometry [17].

### Cell cycle analysis of renal tubular epithelial cells using public scRNA-seq data

We performed cell cycle analysis of tubular epithelial cells using publicly available single-cell RNA sequencing (scRNA-seq) data from the GEO data repository (accession number GSE180420) [18]. The accession number GSE180420 is registered in the Gene Expression Omnibus (GEO), a public repository operated by the National Center for Biotechnology Information (NCBI). This dataset was selected because it employed the same severe ischemia–reperfusion injury model used in our study and compared it with a mild model that recovers spontaneously. The study focused on phenotypic changes in tubular epithelial cells associated with fibrosis progression and successfully identified tubular cell populations specific to the severe model (i.e., conditions with progressive fibrosis), demonstrating reliable model differentiation. We analyzed 23,552 cells from non-injured control mouse kidneys (n = 5) and 35,077 cells from a progressive injury model (Days 1, 3, and 14; n = 2 each). To identify the cell types, we merged and analyzed all kidney samples using anchor-based canonical correlation analysis integrated in the Seurat single-cell analysis package (v.5.1.0) [19]. After normalizing, selecting variable features, and scaling each dataset separately, we performed principal component analysis for dimensional compression. We then used the *IntegrateLayers* function in Seurat to create an anchor and integrate all datasets. Clustering and visualization of the integrated dataset were performed using (uniform manifold approximation and projection). Next, we calculated S and G2/M cell cycle scores using 43 S-phase highly expressed genes and 54 G2/M phase highly expressed gene sets provided by Kowalczyk et al. [20] with the *CellCycleScoring* function in Seurat. Cells with high and positive S scores were classified as in the S phase, those with high and positive G2/M scores as in the G2/M phase, and the remainder as in the G1 phase.

### Statistical analysis

Statistical analyses were performed using JMP statistical software (SAS Institute Inc., NC, United States) and GraphPad Prism (GraphPad Software, San Diego, CA, United States). Statistical methods are described in the respective figure legends.

## Results

**Fig 1:**
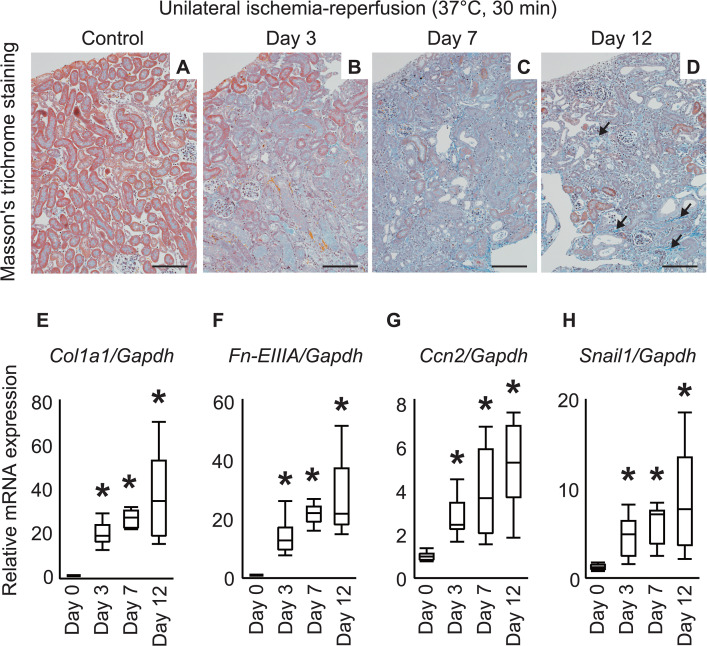
Progressive interstitial fibrosis and increased pro-fibrotic gene expression after unilateral ischemia–reperfusion injury. (A-D) Representation of the Masson’s trichrome staining showing increased collagen fibers (blue) up to 12 days after injury. Conversely, a decrease in the number of normal tubular epithelial cells (red) was observed. Arrows in the Day 12 kidney indicate blue-stained collagen fibers in the interstitium. Furthermore, atrophic tubules characterized by reduced epithelial cell height and sparse brush borders are prominent, representing histological features of chronic kidney disease. Scale bar: 100 μm. (E-H) RT-qPCR analysis showing significant increase in Collagen type I α1 (Col1a1) and fibronectin EIIIA isoform (*Fn-EIIIA*) from Day 3 post-injury, with expression increasing until Day 12. *Ccn2* and *Snail1* expression exhibited similar trends. The relative expression level, with the average expression level of each mRNA on Day 0 (normal kidney before injury), was set to 1.0. Statistical method: Steel’s test (n = 6 per group). * Statistically significant (P < 0.05) versus Day 0. Abbreviations: RT-qPCR, real-time semi-quantitative reverse transcription polymerase chain reaction; *Ccn2*, cellular communication network factor 2; *GAPDH*, glyceraldehyde 3-phosphate dehydrogenase.

Representative kidneys from the UIRI model mice are shown in Fig 1. Masson’s trichrome staining confirmed the progression of interstitial fibrosis and tubular atrophy up to 12 days postoperatively. RT-qPCR results using total RNA from whole kidneys showed a significant increase in extracellular matrix production (Collagen type I α1 [*Col1a1*] and fibronectin EIIIA isoform (*Fn-EIIIA*)) from Day 3–12 after UIRI. In addition to *Col1a1* presented in the results, we also evaluated *Col1a2* expression by RT-qPCR, which showed a similar trend. Furthermore, *Ccn2* showed continued upregulation. *Snail1*, a transcription factor involved in the acquisition of a pro-fibrotic phenotype by tubular epithelial cells, exhibited a similar behavior.

**Fig 2:**
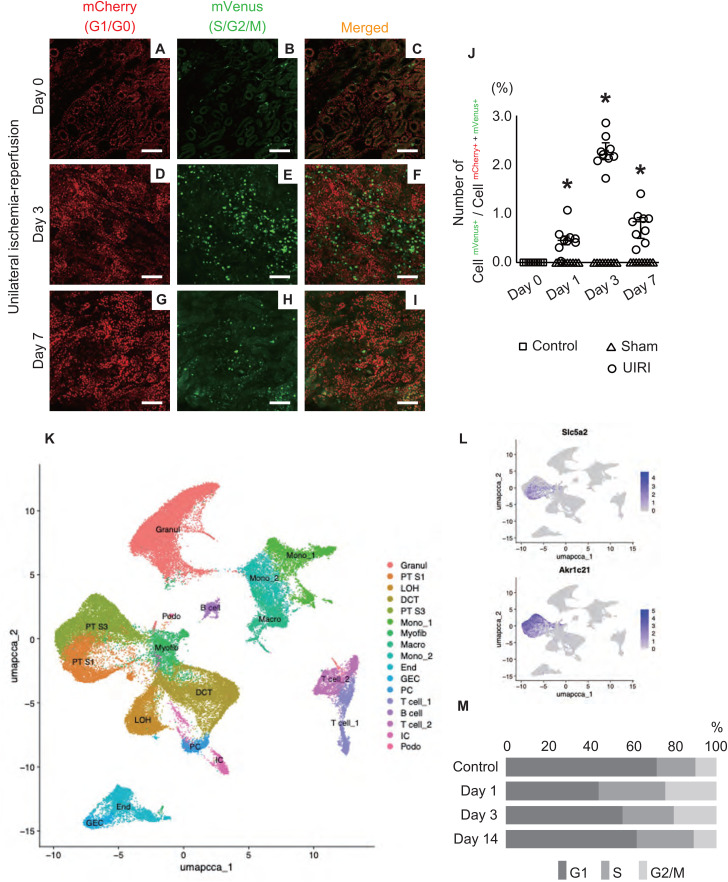
Cell cycle dynamics of tubular epithelial cells after unilateral ischemia–reperfusion injury. (A-I) Representation of fluorescence microscopy images of injured kidney tissue from γGT.Fucci2aR mice. Red fluorescence (mCherry) indicates G1/G0 phase cells, whereas green fluorescence (mVenus) indicates S/G2/M phase cells. Scale bar: 100 μm. (J) Quantification of the ratio of mVenus-positive to mCherry-/mVenus-positive cells in a high-power field (40 × objective lens) used as an index of the proportion of cycling cells. Three mice were used per group, and three tissue sections were prepared and evaluated for each kidney. Control indicates the normal kidney group, Sham indicates the sham-operated group (without renal ischemia treatment), and UIRI indicates the unilateral ischemia–reperfusion injury group. Statistical method: Steel test. * Statistically significant (P < 0.05) versus Day 0. (K-M) Results of integration of single-cell RNA-seq datasets. (K) UMAP visualization of 58,629 cells from mouse kidneys (n = 5 controls and n = 6 injury), colored according to the annotated cell type identity. The cell types were identified using previously described marker genes [18]. The control sample included 10,014 cells derived from PT S1 and S3, and the injury model samples included 2,139 cells of the same cell types. (L) Feature plots of *Slc5a2* and *Akr1c21* genes, markers of PT cells. (M) Changes in the cell cycle at each time point after injury. The proportion of cells in the S phase increased sharply the day after injury; the proportion of cells in the G2/M phase peaked on Day 1 but decreased to levels similar to pre-injury (control) by Day 14. Abbreviations: GEC, glomerular endothelial cell; Endo, endothelial cell; Myofib, myofibroblast; PT S1, proximal convoluted tubule; PT S3, proximal straight tubule; LOH, loop of Henle; DCT, distal convoluted tubule; PC, principal cell; IC, intercalated cell; Granul, granulocyte; Mono, monocyte; Macro, macrophage.

We created a progressive renal injury model in the γGT.Fucci2aR mice to examine the cell cycle dynamics of tubular epithelial cells during CKD progression (Fig 2). In the observation of cleared tissues using the CUBIC method, the proportion of mVenus-positive cells, indicating the S/G2/M phase, showed a transient increase at Day 3 post-surgery and sharp decrease by Day 7 (Fig 2A-2I). Cell counting revealed that although mVenus-positive cells were rarely observed in normal kidneys (S1 Fig), diseased kidneys showed a significant increase in the proportion of mVenus-positive cells from Day 1 post-surgery, reaching a maximum on Day 3, followed by a rapid decrease on Day 7 (Fig 2J). The highest number of fluorescent-labeled positive cells was observed in normal kidneys, with an average of 218 mCherry-positive cells per field. Cell numbers tended to decrease as injury progressed, with the lowest count being an average of 78 cells at Day 7. mVenus-positive cells ranged from 0 to a maximum of 7 cells per field (observed at Day 3).

Furthermore, we validated the cell cycle dynamics of the tubular epithelial cells using publicly available scRNA-seq data [18]. First, we performed clustering analysis based on the gene expression profiles of all cells and visualized them using UMAP dimensionality reduction (Fig 2K). *Slc5a2* or solute carrier family 5 member 2, also known as *SGLT2*, is a sodium-glucose cotransporter predominantly expressed in the S1 segment of the proximal tubule. *Akr1c21* or aldo-keto reductase family 1 member C21 is preferentially expressed in the S3 segment of the proximal tubule. We mapped the expression distribution of *Slc5a2* and *Akr1c21* on UMAP (Fig 2L) and identified the proximal tubular epithelial cell populations through quantitative analysis of *Slc5a2* and *Akr1c21* expression levels in each cluster. For the identified proximal tubular epithelial cell populations, we inferred cell cycle states based on S and G2/M phase scoring. Results revealed that the proportions of both S and G2/M phase cells peaked on Day 1 post-surgery and then gradually decreased toward Day 14, returning to control levels (Fig 2M). These results are consistent with our analysis using the γGT.Fucci2aR mice.

**Fig 3:**
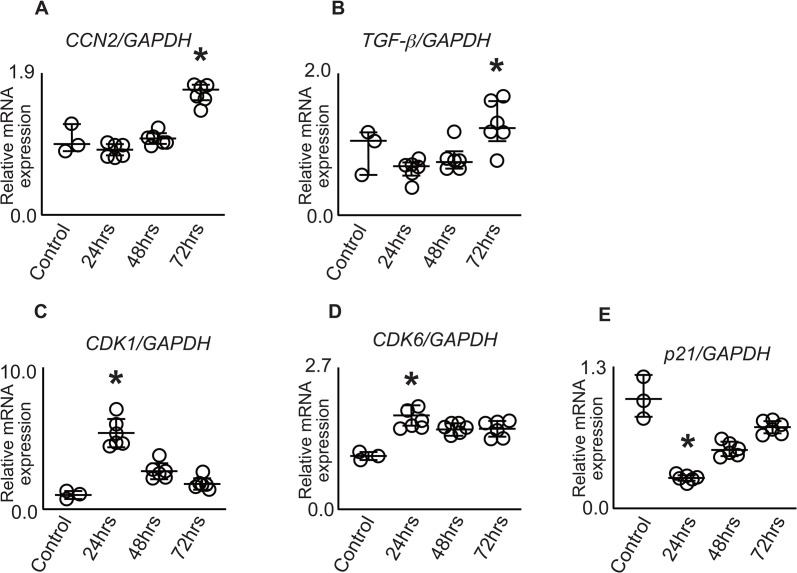
Time-dependent changes in cell cycle-related molecules and pro-fibrotic factors in HK-2 cells under normal culture conditions. (A-E) RT-qPCR analysis showing temporal changes in gene expression. Vertical axis shows the relative expression difference (ΔΔCt value) for each mRNA normalized to glyceraldehyde 3-phosphate dehydrogenase. Values are expressed as fold change relative to the Stnd Cont group (set as 1.0). Time-course expression patterns are shown for (A) *Ccn2*, Cellular Communication Network Factor 2, (B) *Tgf-β*, transforming growth factor-β, (C) *Cdk1*, cyclin-dependent kinase 1, (D) *Cdk6*, cyclin-dependent kinase 6, (E) p21^Waf1/Cip1^. The Wilcoxon test was used to compare the control group to the group with the greatest difference. * Statistically significant (p < 0.05) compared to the control. Abbreviations: RT-qPCR, RT-qPCR, real-time semi-quantitative reverse transcription polymerase chain reaction; HK-2, human kidney-2.

Furthermore, we analyzed the relationship between the cell cycle and the gene expression of pro-fibrotic factors using human renal proximal tubular epithelial cells (HK-2). When cells cultured under low-oxygen and low-nutrient conditions (Control) were transferred to normal culture conditions (growth medium), the expression of CCN2 and TGF*-*β increased up to 72 h (Fig 3A, 3B). Cyclin-dependent kinase (CDK) 1 is essential for G2/M transition and mitotic entry, and its expression indicates cell progressing through cell division. CDK6 promotes G1/S transition by phosphorylating retinoblastoma protein, facilitating cell cycle progression from G1 to S phase. p21^Waf1/Cip1^, also known as CDKN1A, is a cyclin-dependent kinase inhibitor that binds to and inhibits cyclin-CDK complexes, thereby inducing cell cycle arrest at both G1/S and G2/M checkpoints. In the control cells (under low-oxygen and low-nutrient conditions), the expression of *CDK1* and *CDK6* was low, while *p21*^*Waf1/Cip1*^ showed high expression, suggesting that the cell cycle was synchronized in the quiescent phase (Fig 3C-3E). Upon transfer to normal culture conditions, rapid upregulation of *CDK1* and *CDK6* expression and downregulation of *p21*^*Waf1/Cip1*^ expression were observed. After 72 h of culture, *CDK6* gene expression was maintained, *CDK1* gene expression decreased over time, and *p21*^*Waf1/Cip1*^ expression gradually increased, suggesting cell cycle arrest. These results indicate that cell cycle regulators function appropriately and maintain the cycle during proliferation and arrest. Notably, *CCN2* and *TGF*-β expression could be induced, even when the cell cycle was in a normal state.

**Fig 4:**
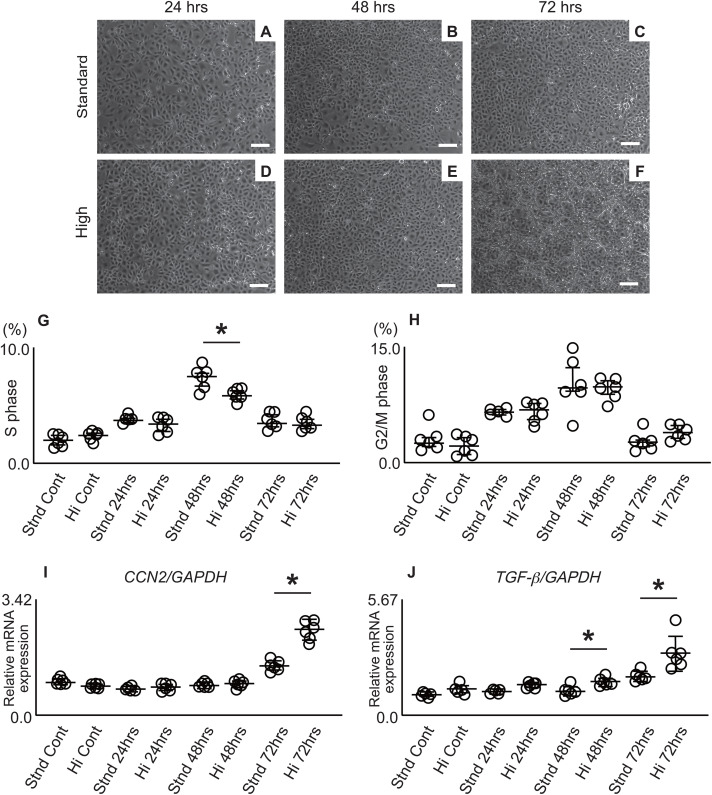
Effects of cell density on cell cycle progression and pro-fibrotic factor expression in HK-2 cells. (A-F) Phase-contrast microscopy images showing cell morphology under standard and high-density conditions. Images were captured at 24 h (A, D), 48 h (B, E), and 72 h (C, F) after seeding. Upper panels (A-C) show standard seeding density conditions (Standard), and lower panels (D-F) show high-density conditions (Hi). Scale bar: 200 μm. (G, H) Flow cytometric analysis of cell cycle distribution showing the percentage of cells in the (G) S and (H) G2/M phases at different densities. (I, J) Time-course expression patterns determined by RT-qPCR for (I) CCN2, cellular communication network factor 2, and (J) TGF-β, transforming growth factor-β. The vertical axis shows the relative expression difference (ΔΔCt value) for each mRNA normalized to glyceraldehyde 3-phosphate dehydrogenase. Values are expressed as fold change relative to the Stnd Cont group (set as 1.0). Statistical methods: The Wilcoxon test was performed for pairwise comparisons at each time point. * Statistical significance (p < 0.05) between the Standard (Stnd) and Hi conditions. Cont, Control.

We focused on the cell number on the culture dish as a potential cause for the differential expression of CCN2 and TGF-β between “Control” and “72 h,” both of which showed similar quiescent cell cycle states. Hypothesizing that this difference in cell number was related to the expression status of *CCN2* and *TGF-β*, we examined the effect of cell number/culture environment on the expression of pro-fibrotic factors (here, CCN2 and TGF-β) by setting two seeding conditions: “standard cell number (Standard: Stnd)” and “high cell number (High: Hi),” which was twice the standard. In the standard cell number group, the cells reached 100% confluence at 72 h, displaying a characteristic cobblestone-like arrangement of tubular epithelial cells. In contrast, the high cell number group reached 100% confluence at 48 h, and by 72 h, morphological changes due to overcrowding (spindle-shaped transformation and partial lifting) were observed (Fig 4A-4F).

Next, we analyzed actual cell cycles using flow cytometry under standard and high-density conditions (Figs 4G and 4H).

Flow cytometry measurements of S and G2/M phase cells showed an increase from 24 to 48 h, confirming cell cycle progression, as expected. At 48 h, the high-density condition (Hi) tended to have a lower proportion of S-phase cells than the standard condition (Stnd), suggesting that cell progression slowed slightly earlier under high-density conditions. At 72 h, the proportion of cells in the S and G2/M phases decreased again under both conditions and returned to levels similar to those of the control, confirming a cell cycle slowdown.

*CCN2* expression under these conditions increased over time, peaked at 72 h, similarly to that in Fig 3. However, the high-density group (Hi) showed significantly higher *CCN2* expression than the standard condition group (Stnd). A similar trend was observed for TGF-β, which also peaked at 72 h, with significantly higher expression under high-density conditions (Hi) than standard conditions (Stnd) (Figs 4I and 4J).

## Discussion

This investigation of the relationship between the cell cycle of tubular epithelial cells and their pro-fibrotic phenotype showed that cells in the G2/M phase were a minor population, even during progressive interstitial fibrosis. Instead, as in normal kidneys, many tubular epithelial cells in fibrotic kidneys were observed in the G1/G0 phase. In cultured cells using *CCN2* and *TGF-β* expression as markers of a pro-fibrotic phenotype, expression was significantly increased in cell populations whose cell cycle was slowing and were attempting to re-enter G1/G0 arrest as cell density increased after normal passaging, particularly under overcrowded conditions.

CCN2 is a matricellular protein, whose expression increases during fibrosis in various organs. In the kidney, CCN2 binds to integrins in tubular epithelial cells and promotes renal fibrosis [3]. In our model, Snail1, a representative transcription factor involved in the phenotypic changes of tubular epithelial cells [21–23], showed a similar increase in expression to extracellular matrix components and CCN2, confirming that our mouse model exhibited progressive renal fibrosis and phenotypic changes in tubular epithelial cells that are likely involved in fibrosis. Although our study focused on tubular epithelial cells as CCN2-expressing cells, other cell types in fibrotic kidneys can also express CCN2. However, it should be noted that knockdown of CCN2 specifically in tubular epithelial cells significantly reduces overall CCN2 expression in the kidney and attenuates fibrotic lesions [2].

Although studies using Fucci2aR stable expression cultured cells and anticancer agents have confirmed that G2/M-arrested cells appear green (mVenus expression) and G1-arrested cells appear red (mCherry expression) [24], whole-mount examination of embryos during development showed that the red color intensifies not only in G1 arrest but also in the G0 post-mitotic phase [25]. Additionally, in cultured cells (tetraploids) with impaired division due to high concentrations of etoposide, changes from green to red without cell division have been observed during formation [24]. Recent studies have reported the existence of alternative cell cycles that generate polyploid proximal tubular cells [10,26,27]. Therefore, it is difficult to obtain a complete picture of abnormal cell cycling using fluorescent markers. Nevertheless, we believe that our G2/M arrest evaluation results are reliable because the G2/M-arrested states were observed as yellow-green to green with mVenus expression.

Studies using proliferating cell nuclear antigen staining reported that the number of cells progressing through the cell cycle after ischemia–reperfusion injury was highest at 3 days after injury [28]. Our model showed similar results, with mVenus-positive cells being most abundant at 3 days after injury and decreasing rapidly by Day 7. These G2/M-arrested cells stop at the G2/M checkpoint because of severe genetic damage [8]. Considering this mechanism, an increase after Day 7 was unlikely. Accordingly, the estimated maximum proportion of mVenus-positive cells was 2–3%, which is reasonably consistent with the proportion of cells positive for phosphorylated histone H3 at serine 10 residue in previous studies [2,29]. Because our examination using γGT.Fucci2aR mice and tissue sections might have underestimated the number of G2/M cells, we performed cell cycle analysis using public scRNA-seq data [18]. Our evaluation of the dynamic nature of tubular epithelial cell cycles in the acute phase after injury was appropriate, showing that the proportion of G2/M phase cells in the chronic phase of the progressive model was almost the same as that in normal kidneys.

Based on our finding of abundant G1/G0 phase cells during fibrosis progression in mouse models of CKD, we developed an *in vitro* model using cultured cells. As cell cycle-related molecules, we examined CDK6, which is related to G1 phase progression; CDK1, related to G2 phase progression and M phase initiation; and p21^Waf1/Cip1^, widely recognized as a CDK inhibitor associated with cell cycle arrest [10]. The results suggested a course of cellular proliferation, namely a gradual increase in density, eventual confluence, and then a re-slowing of the cell cycle, as confirmed by flow cytometry. Thus, tubular epithelial cells in G1/G0 can express CCN2 under appropriate conditions (overcrowding) and stimulation (TGF-β). Immediately after resting treatment, G1/G0 phase-quiescent cells showed low levels of TGF-β and CCN2 expression, indicating that their expression is not solely dependent on cell cycle state.

The mechanism of the cell density-mediated suppression of proliferation is called contact inhibition. TGF-β and insulin-like growth factor binding protein-6 (IGFBP-6) in culture supernatant have been identified as related humoral factors [30]. TGF-β induces cell cycle arrest in G1 by inhibiting CDK4/6 through the induction of p21 expression [31,32]. CCN2 is also highly expressed under several types of mechanical stress [33,34], suggesting that overcrowding itself may have induced CCN2 expression. Regarding the cultured cells, we cannot exclude the possibility that immortalization effects or culture-related stresses such as overcrowding, which are not directly related to cellular damage, may have contributed to the induction of CCN2 expression. Recent reports have also suggested that the Hippo pathway and hypoxic conditions are involved in inducing CCN2 expression in the kidney [35].

Our study has some limitations that should be acknowledged. Although our protocol was designed to mimic mouse ischemia–reperfusion, we could not mimic the DNA damage to tubular epithelial cells expected in severe renal injury. When the cell damage was too severe, the subsequent induction of CCN2 expression was unsuccessful. Therefore, we will conduct further experiments to investigate these aspects. To examine the effects of CCN2 and TGF-β expressed by tubular epithelial cells on extracellular matrix production under conditions more closely resembling the *in vivo* environment, evaluation using more refined culture systems, such as co-culture with fibroblasts (with ascorbic acid supplementation), may be worth considering in future studies.

## Conclusion

Tubular epithelial cells may exhibit a pro-fibrotic phenotype, not only during G2/M phase arrest. The development of therapeutic drugs for fibrosis/chronic kidney disease that target the abnormal cell cycle of tubular epithelial cells requires a comprehensive approach, including G1 phase cell arrest and polyploid formation.

## Supporting information

S1 Fig Fluorescence microscopy findings. Fluorescence microscopy images of control (normal kidney) from γGT.Fucci2aR mice.
(EPS)
